# Using Novel Molecular-Level Chemical Composition Observations of High Arctic Organic Aerosol for Predictions of Cloud Condensation Nuclei

**DOI:** 10.1021/acs.est.2c02162

**Published:** 2022-09-16

**Authors:** Karolina Siegel, Almuth Neuberger, Linn Karlsson, Paul Zieger, Fredrik Mattsson, Patrick Duplessis, Lubna Dada, Kaspar Daellenbach, Julia Schmale, Andrea Baccarini, Radovan Krejci, Birgitta Svenningsson, Rachel Chang, Annica M. L. Ekman, Ilona Riipinen, Claudia Mohr

**Affiliations:** † Department of Environmental Science, Stockholm University, Stockholm SE-10691, Sweden; ‡ Department of Meteorology, Stockholm University, Stockholm SE-10691, Sweden; § Bolin Centre for Climate Research, Stockholm University, Stockholm SE-10691, Sweden; ∥ Department of Physics and Atmospheric Science, Dalhousie University, Halifax CA-B3H 4R2, Canada; ⊥ Laboratory of Atmospheric Chemistry, Paul Scherrer Institute, Villigen CH-5232, Switzerland; # Extreme Environments Research Laboratory, École Polytechnique Fédérale de Lausanne, Sion CH-1951, Switzerland; ∇ Division of Nuclear Physics, Lund University, Lund SE-22100, Sweden

**Keywords:** aerosol−cloud interactions, cloud droplet activation, CCN closure, atmospheric aerosol, aerosol chemistry, chemical ionization mass spectrometry (CIMS), High Arctic

## Abstract

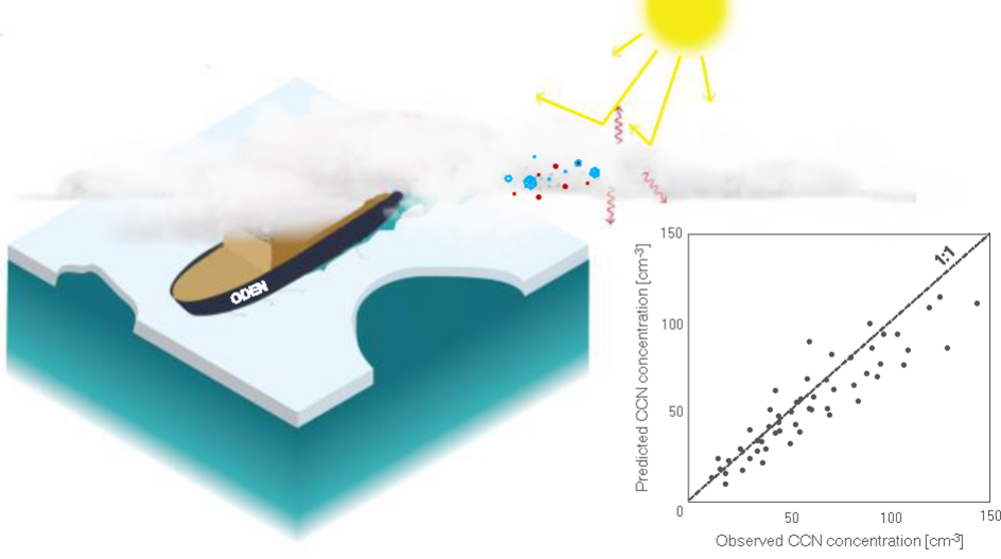

Predictions of cloud droplet activation in the late summertime (September) central Arctic Ocean are made using *κ*-Köhler theory with novel observations of the aerosol chemical composition from a high-resolution time-of-flight chemical ionization mass spectrometer with a filter inlet for gases and aerosols (FIGAERO-CIMS) and an aerosol mass spectrometer (AMS), deployed during the *Arctic Ocean 2018* expedition onboard the Swedish icebreaker *Oden*. We find that the hygroscopicity parameter *κ* of the total aerosol is 0.39 ± 0.19 (mean ± std). The predicted activation diameter of ∼25 to 130 nm particles is overestimated by 5%, leading to an underestimation of the cloud condensation nuclei (CCN) number concentration by 4–8%. From this, we conclude that the aerosol in the High Arctic late summer is acidic and therefore highly cloud active, with a substantial CCN contribution from Aitken mode particles. Variability in the predicted activation diameter is addressed mainly as a result of uncertainties in the aerosol size distribution measurements. The organic κ was on average 0.13, close to the commonly assumed *κ* of 0.1, and therefore did not significantly influence the predictions. These conclusions are supported by laboratory experiments of the activation potential of seven organic compounds selected as representative of the measured aerosol.

## Abstract 2

Predictions of cloud formation need to be improved for future climate projections. This study from the Arctic shows how detailed chemical information on the atmospheric aerosol affects the prediction accuracy.

## 1 Introduction

Aerosol–cloud interactions are associated with large uncertainties in projections of past and future climates.^1^ These uncertainties also affect our understanding of *Arctic amplification*, that is, the high current annual average surface level warming in the Arctic region (two to four times higher than the global average of +1 °C compared to preindustrial times).^2^ This phenomenon is a result of several remote and local processes and feedbacks, which to date are not fully understood.^3^ More detailed experimental data on aerosols and clouds, especially for the High Arctic (>80° N), where direct observations are scarce, are needed to decrease the uncertainties.^4^

The potential for aerosol particles to activate as cloud droplets can be estimated using *κ*-Köhler theory, which is a semiempirical theory based on simplified equilibrium thermodynamics. It describes the saturation ratio of water vapor over an aqueous droplet of a certain size and composition.^5,6^*κ* (kappa) is the compound- and mixture-specific hygroscopicity parameter. *κ*-values range from 0 for completely nonhygroscopic aerosol particle components (*e.g.*, soot)^7^ to about 1.5 for very hygroscopic components (*e.g.*, sodium chloride, NaCl).^8^ Most of the oxygen-containing organic aerosol compounds have *κ*-values in the range of 0.01–0.3,^9^ but they can reach as high as 0.4 in some areas of the world.^10^*κ* can be determined either from experiments or theory.^11,12^ With *κ*-Köhler theory, the number of aerosol particles that get activated as cloud droplets (cloud condensation nuclei, CCN) can be estimated if the aerosol particle composition and size distributions are known, under the assumption of an internally mixed aerosol. Comparisons of such calculations to measured CCN concentrations yield insights into the factors controlling aerosol–cloud interactions, and such “CCN closure” approaches have been applied at several locations around the world,^13−15^ including the High Arctic.^16^ The latter treated the aerosol particles as if they were composed of sulfate and organic compounds only, assuming different possible combinations of *κ*-values and soluble organic fraction, without knowing further details about the composition. They found that the CCN number concentration was overpredicted in most of the cases and that the highest closure was achieved when a nearly water-insoluble organic fraction was assumed. Although the authors could not fully explain this overprediction with their data set, they concluded that more detailed aerosol chemical composition data could be a step toward further insights.

In the High Arctic, aerosol sources largely vary with season.^17−19^ Earlier observations during summertime have shown that sea spray aerosol (SSA), which is a combination of inorganic sea salt and organic matter,^20,21^ is an important source of aerosol particles to the Arctic Ocean^22,23^ marine boundary layer. The inorganic fraction of the aerosol mass is already relatively well understood as it is composed of ionic salts such as NaCl. The organic fraction on the other hand is a complex mixture of molecules with a large variation in composition and chemical properties from a variety of different sources,^24−26^ including compound classes such as proteins,^27^ polysaccharides,^28,29^ lipids,^30^ oxidation products of dimethyl sulfide,^31,32^ and water-insoluble marine polymer gels^33−35^ largely produced by microbiological activity in the ocean. The biogenic material is accumulated in the sea surface microlayer (SML) and is emitted as primary aerosol particles to the atmosphere through bubble bursting at the ocean surface.^36,37^

In our previous work,^38^ we presented the composition of secondary submicron (particle diameter < 1 μm) aerosol with unprecedentedly high chemical resolution of the organic fraction sampled in the Arctic Ocean in September 2018. In thecurrent study, we use these results together with *in situ* measurements of aerosol size distributions, to estimate CCN concentrations and activation diameters at varying supersaturations for the central Arctic Ocean boundary layer. The estimates are compared to direct observations of CCN. Furthermore, the aerosol hygroscopicity in the real Arctic atmosphere is compared to results from laboratory experiments of the cloud droplet activation potential of a range of organic species. As such, this study adds to previous knowledge^16,39,40^ about CCN and cloud formation in the High Arctic.

## 2 Methods

### 2.1 Microbiology-Ocean-Cloud-Coupling in the High Arctic (MOCCHA) Campaign

The results in this study are based on collected samples and *in situ* measurements from the MOCCHA campaign, part of the research expedition *Arctic Ocean 2018* with the Swedish Icebreaker *Oden*.^41^ The campaign took place from August 1 to September 22, 2018. The geographical locations for the scientific activities ranged from the marginal ice zone (MIZ, ∼82° N) north of Svalbard to close to the North Pole (89° N), where the icebreaker was moored and drifting for nearly 5 weeks. The general goal of MOCCHA was to investigate potential links between marine microbiology, local aerosol emissions, and cloud formation in the central Arctic Ocean.^29,38,42−48^

### 2.2 Aerosol Sampling and Characterization

In this section, we give a brief summary of the experimental setup, sampling conditions, and data analysis of the aerosol samples used for this study. This information has previously been described in the study by Siegel *et al*.^38^ We refer to that publication for further details.

Between September 11 and September 19, during the autumn freeze-up, 13 polytetrafluoroethylene (PTFE) filter samples (referred to as **F1**-**F13**, out of which **F6** was not analyzed)^38^ were collected behind a whole-air inlet (no particle diameter cut-off) located at 25 m above sea level (4^th^ deck of *Oden*) for offline analysis with a high-resolution time-of-flight chemical ionization mass spectrometer with a filter inlet for gases and aerosols (FIGAERO-CIMS).^49^ The analysis provided chemical information at a molecular level on the semivolatile fraction (evaporating at ≤200 °C) of the aerosol, which excludes compounds such as inorganic salts^50^ and likely also marine gels.^51^ The reagent ion deployed in the FIGAERO-CIMS for this study was iodide (I^–^), which clusters predominantly with polar, oxygenated compounds and is less sensitive to hydrocarbons, monoalcohols, and other compounds with a low degree of oxygenation.^52^ The data set can be found on the Bolin Centre Database.^53^

The FIGAERO-CIMS data were supported by measurements made onboard *Oden* with a high-resolution time-of-flight aerosol mass spectrometer (AMS),^54,55^ used to measure mass concentrations of nonrefractory inorganic (sulfate: SO_4_^2–^, nitrate: NO_3_^–^, ammonium: NH_4_^+^, and chloride: Cl^–^) and organic (Org) compounds in the size range of ∼80 nm–1 μm^54,56,57^ and to calculate their relative contributions for each FIGAERO-CIMS filter sample. A multiangle absorption photometer (MAAP)^58^ was used to quantify equivalent black carbon (eBC)^59^ and a differential mobility particle sizer (DMPS) in connection to a WELAS aerosol spectrometer^60,61^ to provide particle number size distributions between 10 nm and 9.65 μm. The number of ambient aerosol particles that activated into cloud droplets (where the droplets were in the size range of 0.75–10 μm) was measured using a cloud condensation nuclei counter (CCNC).^62^ More details of these instruments and the following data analysis are found in Section S1 in the Supporting Information.

### 2.3 Laboratory Experiments on the CCN Activation Potential of Organic Compounds Found in Arctic Aerosol

To add support to the findings in the field, the cloud droplet activation potential of seven organic compounds (levulinic acid, succinic acid, undecanoic acid, glucose, lactose, sodium alginate, and alanine) and sea salt was measured in laboratory experiments. The organic compounds were selected to represent a range of molecular properties, including those of species known to be present in the High Arctic summertime aerosol from previous studies^8,18,20,22^ and our results from the MOCCHA campaign with molecular-level chemical information.^38^ The measured hygroscopicity parameter (referred to as *κ*_lab_) from the compounds in the laboratory study served as a comparison to the hygroscopicity parameters (*κ*_MS_, where MS stands for mass spectrometer, see Section 2.4) of the particles observed during MOCCHA. The laboratory experiments are described further in the Supporting Information (Section S2, Table S1 and Figures S1–S3).

### 2.4 Köhler Calculations

#### 2.4.1 Parameters of the Chemical Composition Data

*κ*-Köhler theory was used to predict the number concentration of CCN and activation diameter based on the aerosol chemical composition information and size distribution data. The procedure for this data analysis is described below.

The maximum *κ*-value of a compound in an ideal solution can be calculated directly from eq 1 as a function of the relationship between the molecular weight and density of water (*M*_w_, *ρ*_w_) and the molecular weight and density of the fully dissolved (into *n* ions) compound (*M*_s_, *ρ*_s_):^6^
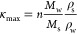
1

Eq 1 was used to get an estimate of the *κ*-values of the organic aerosol fraction (assuming *n* = 1) measured in the High Arctic during MOCCHA and of nitric acid (HNO_3_, assuming *n* = 2) and hydrochloric acid (HCl, assuming *n* = 2), for which no published *κ*-values were found in the literature. As an approximation of *ρ*_s_ of the organic fraction (*ρ*_org_), the density of *β*-caryophyllene secondary organic aerosol (SOA) of 1.22 g cm^–3^ was used. This value is considered to be representative of more complex SOA with a larger number of carbon atoms^63^ and hence also the compounds measured by FIGAERO-CIMS in the Arctic. *M*_s_ of the organic fraction (*M*_org_) was calculated from the median *M*_s_ of the organic compound classes *CHO*, *CHON*, *CHONS*, and *CHOS*, based on their relative contributions to each filter sample **F1**-**F13** measured by FIGAERO-CIMS (Table S2). Because only relative contributions were retrieved from the FIGAERO-CIMS,^38^ they were scaled to the well-quantified Org fraction measured by AMS, meaning that the FIGAERO-CIMS data were used to represent the organic mass fraction in its entirety. A discussion on the limitations of this assumption and descriptions of other parameters used for the calculations are found in Section S3.1 and Table S3.

The organic *κ* (*κ*_org,MS_) was calculated for each filter sample **F1-F13** with eq 1 based on *M*_org_ of each filter sample and *ρ*_org_. Total *κ*_tot,MS_-values of the bulk aerosol were calculated as a mass-weighted average^6^ of the *κ*_s,MS_ of the different organic and inorganic species using eq 2:
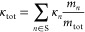
2For  = {organic compounds of each filter sample, SO_4_^2–^, NO_3_^–^, NH_4_^+^, Cl^–^, eBC}, where *m* is the mass concentration, and *κ*_*n*_ is the *κ*_s,MS_-values from eq 1. Because of the small production of ammonium (NH_4_^+^) in the central Arctic Ocean^64^ and its seemingly low contribution to the submicron aerosol mass, as seen in the AMS data and also supported by previous studies,^24,42^ the measured aerosol in the High Arctic was assumed to be very acidic. Although NH_4_^+^ was detected in ultrafine aerosol particles (*D*_p_ = 20–60 nm) during the expedition,^29^ the overall contribution of NH_4_^+^ to the total submicron aerosol mass is negligible, and SO_4_^2–^, Cl^–^, and NO_3_^–^ were assumed to be mainly present as acids instead of ammonium salts. This and the origin of measured black carbon are discussed further in Section S3.2.

*κ*_tot,MS_ was calculated for two different cases as an investigation of the importance of time resolution of the chemical composition data for CCN closure. For the first case, referred to as **FC-tr** (FIGAERO-CIMS time resolution = 1 value per filter), we used the median *m_n_* and *κ*_*n*_ of each filter sample (Table S2) to calculate a *κ*_tot,MS._ In the second case, called **AMS-tr** (AMS time resolution = 5 min), we instead used *m_n_* and *κ_n_* of every time step in the AMS data (where *κ*_org,MS_ of the respective filter sample was used for the organic fraction) to calculate a *κ*_tot,MS_. Hence, the **FC-tr** case resulted in 12 *κ*_tot,MS_-values and **AMS-tr** in 1286 *κ*_tot,MS_-values, which were averaged to the filter sampling periods for comparison to **FC-tr**. A graphical example of this is shown in Table S4. Values of *M*_s_, *ρ*_s,_ and *κ* used for the calculations are listed in Table S5.

#### 2.4.2 Prediction of CCN Concentration

For the bottom-up prediction of the activation diameter and CCN number concentration based on *κ*-Köhler theory, we assumed that all particles of a given size measured by the DMPS+WELAS are equally able to activate. We also assumed an internally mixed homogeneous composition throughout the size distribution (as seen previously for marine aerosols in remote oceans^65^ and in a CCN closure study of sub-Arctic aerosol, where the predictions were equally strong when using an internally and externally mixed aerosol^66^), and in practice a fully soluble organic fraction that did not affect the surface tension to a significant degree. This is a simplification, as our previous study indicated the presence of nonsoluble organic compounds such as long-chain fatty acids,^38^ which in theory could lower the surface tension and hence increase the CCN activation potential.^67^

The procedure for the prediction analysis is illustrated by an example in Figure S4. First, the activation diameter of the particles measured by the CCNC (*D*_p,act,obs_) was found by matching the measured CCN number concentration (*CCN*_obs_) with the corresponding particle number concentration in the cumulative number size distribution, starting from the largest diameter. This provided an activation diameter for the CCNC observations (*D*_p,act,obs_). *κ*-values of each CCNC time step (*κ*_CCNC_) were then determined through a rearrangement of eq 3,^68^ where the critical supersaturation (*SS*_crit_) was set to the supersaturation (*SS*) of the CCNC. To find the predicted activation diameter (*D*_p,act,pred_), *SS*_crit_ was calculated by *κ*-Köhler theory through eq 3 for each diameter in the size distribution data and *κ*_tot,MS_-value:
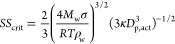
3where *D*_p,act_ is the dry particle activation diameter in the size distribution data, *R* is the ideal gas constant, *T* is the temperature of the CCNC inlet manifold, and *M*_w_, *ρ*_w,_ and *σ* are the molar mass, density, and surface tension of water, respectively.

The *SS* in the CCNC was then matched with these calculated *SS*_crit_ values to find the corresponding *D*_p,act,pred_ at each time step. The cumulative particle concentration at these critical diameters was then assigned as the predicted CCN number concentration (*CCN*_pred_). For further analysis, we set some constraints on the data set for what was considered to be useful data. These are summarized in Section S4.1.

## 3 Results and Discussion

### 3.1 Aerosol Chemical Composition

The molecular composition of the aerosol particles used for this study is already discussed in detail in the study by Siegel *et al*.^38^ and only a brief summary will be given here.

In total, we detected 519 compounds clustered with I^–^ that were above the limit of detection. The detected organic compounds were grouped into four categories depending on the atoms included in the molecular composition, *CHO*, *CHON*, *CHONS*, and *CHOS* (C standing for carbon, H: hydrogen, O: oxygen, N: nitrogen, and S: sulfur). The largest contribution was from *CHO* and *CHON* compounds (98% by mass), with an average number of 9 C atoms and an average oxygen-to-carbon (O:C) ratio of ∼0.65. *CHONS* and *CHOS* compounds were not as prevalent and had a lower average C number (4), but a higher O:C ratio (∼1.3) compared to the *CHO* and *CHON* compounds. Overall, the most common numbers of O atoms were 3–4, but there was also a pronounced contribution of compounds with a high carbon number (>11) together with a low oxygen number (1–2), with molecular formulae corresponding to long-chain fatty acids.

### 3.2 CCN Activation Potential during the Sampling Period

**Figure 1:**
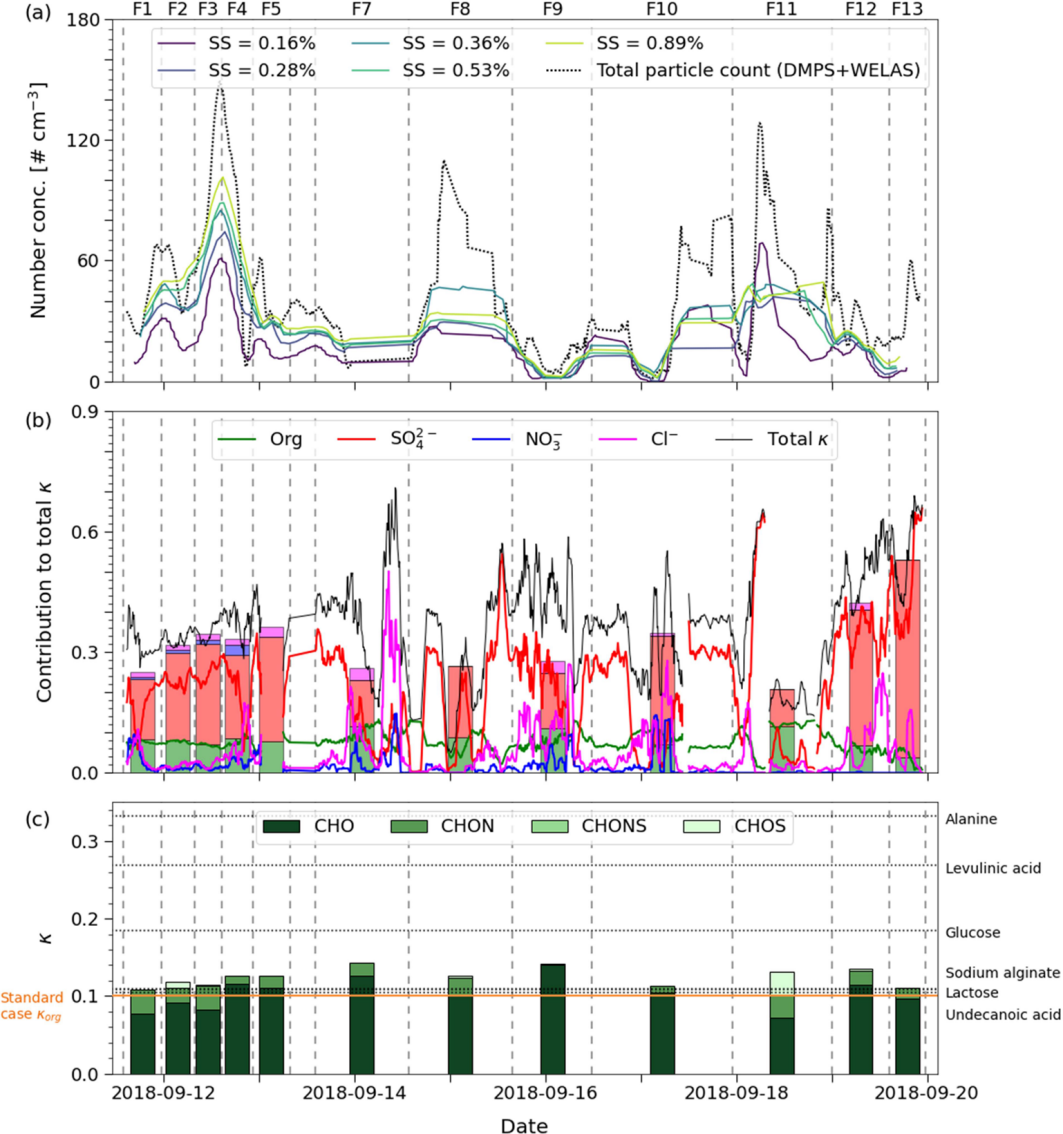
Time series of: (a) running average (90 min) of the total particle number concentration in the size range of 10 nm–9.7 μm and measured CCN number concentrations at different supersaturations (*SS*). Vertical lines show the start and end times of each FIGAERO-CIMS filter sample and filter numbers are written above the figure; (b) relative contributions of the AMS species to the total *κ*-value of each filter sample (*κ*_tot,MS_). The bars are stacked, where *κ*_tot,MS_ of the **FC-tr** case is represented by the bar height, and the contribution of each AMS species by the colored areas. The black line represents *κ*_tot,MS_ of the **AMS-tr** case and the colored lines the contribution of each species (running average of 90 min, where gaps in the time series are due to missing data); (c) *κ*_org,MS_ divided into FIGAERO-CIMS organic compound classes (*CHO*, *CHON*, *CHONS*, and *CHOS*, meaning molecules containing carbon, hydrogen, oxygen + nitrogen, and/or sulfur), scaled to AMS Org. The dotted lines show *κ*_lab_ of the organic compounds in the laboratory study (where succinic acid was left out because of questionable results, see Figure S6) and the orange solid line the standard case of *κ*_org_ = 0.1 as comparison to the *κ*_org,MS_ values.

Overall, the aerosol particles measured during the nine sampling days (September 11–19, 2018) exhibited, at *SS* = 0.38%, a CCN activation ratio of 0.44 ± 0.28 (mean ± std) of the total number of ambient aerosol particles >10 nm (Figure 1a). This is relatively similar compared to the average of ∼0.50 (from the linear fit equation at *SS* = 0.38%)^69^ at a remote and marine North Atlantic site with a relatively higher contribution of sulfate to organics^70^ and ∼0.40 ± 0.15 (*SS* = 0.40%) at an urban site in China.^71^ It is however considerably higher than the annual average of 0.13 at *SS* = 0.50% in Vienna, Austria,^72^ where the aerosol is characterized as well-mixed urban background aerosol. In the study in China, the aerosol contained high amounts of inorganics (average 76.2%) compared to measurements in European cities (average ∼ 35%),^73^ which is probably an explanation for the high hygroscopicity in China. Considering that the aerosol organic/inorganic ratio in this study was more similar to the samples in Europe, the Arctic aerosol must be considered to be highly hygroscopic. The assumption that all particles of a certain size were equally able to activate (see Section 2.4.2) does hence appear to be valid.

During the periods when *Oden* was moored to an ice floe (samples **F1**-**F7** and **F12**-**F13**), the activation ratio was more stable compared to the transit period (**F8**-**F11**). Despite efforts to remove periods of possible contamination from the ship stack for these samples (when the pumps for the FIGAERO-CIMS filter sampler were off), the variation in particle concentration was larger in these samples compared to **F1**-**F7** and **F12**-**F13**, especially at smaller particle diameters. In addition, periods with elevated risk of contamination (identified based on particle number concentration/distribution and BC measurements^38^) were removed from the data set, which caused a more scattered time series compared to the ice floe samples. These are the reasons why the running averages of CCN number concentrations in Figure 1a sometimes appear to be higher at lower *SS* levels and vice versa.

Figure 1b shows the relative contribution to *κ*_tot,MS_ of inorganic and organic species during the filter sampling times measured by AMS and FIGAERO-CIMS (mass contributions to each sample are shown in Figure S5 and Table S6). Overall, SO_4_^2–^ contributed most to *κ*_tot,MS_ followed by Org, which remained relatively stable throughout the sampling period but decreased somewhat in the MIZ (September 19). Despite the low mass contributions of Cl^–^ and NO_3_^–^, they have an apparent contribution to *κ*_tot,MS_ due to their high individual*κ*-values, especially when the contribution from SO_4_^2–^ was low (*e.g.*, September 14 and 17). The highest *κ*_tot,MS_ of the filter samples was found in the MIZ samples **F12** and **F13**, where SO_4_^2–^ had a much higher relative contribution (80–93%) compared to the samples from the ice floe (**F1**-**F7**, 44–72%) and the transit (**F8**-**F11**, 45–78%). **F8** had a considerable amount of eBC, which reduced the overall hygroscopicity of this sample. The level of eBC was negligible in the other transit samples (Table S6).

Figure 1c shows the *κ*_org,MS_ absolute values of the filter samples with contributions from the FIGAERO-CIMS organic classes *CHO*, *CHON*, *CHONS*, and *CHOS* scaled to AMS Org. The sulfur-containing classes *CHONS* and *CHOS* had overall higher *κ*-values (mean 0.19 and 0.16, respectively) compared to *CHO* and *CHON* (mean 0.13 and 0.11, respectively). Despite this, the most influential organic compound class was *CHO* followed by *CHON* because of their higher mass loadings. The dotted horizontal lines in Figure 1c serve as a comparison of *κ*_org,MS_ and the *κ*_lab_ of the organic compounds from the laboratory study (succinic acid was left out because of a complex activation pattern^74^ that led to questionable results, see Figure S6). The *κ*_lab_ values are tabled in Table 1 together with an explanation of what each substance could represent in the Arctic aerosol. Alanine, levulinic acid, and glucose were more hygroscopic than the average field sample, whereas sodium alginate, lactose, and undecanoic acid were at a similar level to the field samples. This means that a larger contribution of compounds with similar chemical properties to alanine, levulinic acid, and glucose could increase the *κ*_org,MS_. Adding sodium alginate, which is supposed to represent marine gels which accumulate in the SML, would on the other hand not increase *κ*_org,MS_ substantially. However, an earlier chamber study showed that the hygroscopicity of aerosol particles generated from desalted High Arctic SML samples was very high (*κ* ∼ 1),^75^ implying that sodium alginate is not fully representative of these aerosols. The orange line in Figure 1c corresponds to the commonly assumed *κ*_org_ of 0.1 in calculations and models.^14,76−78^ We call this the “standard case.” Our filter samples were hence slightly more hygroscopic than the standard case, however, rounded to the same number of significant digits (1) they would all be 0.1.

**Table 1:** Experimentally Determined κ-Values (κ_lab_) of Compounds Thought to Be Representative of Submicron Summertime Central Arctic Aerosol^a^

Compound	Molecular formula	Compound class	*κ* (1 std)
sea salt	mix of inorganic ions^b^	inorganic salt mixture^22,23^	1.14 (0.072)
levulinic acid	C_5_H_8_O_3_	compound with three oxygen atoms^38^	0.268 (0.007)
succinic acid^c^	C_4_H_6_O_4_	compound with four oxygen atoms^38^	0.127 (0.072)
undecanoic acid	C_11_H_22_O_2_	long-chain fatty acid^30,38^	0.104 (0.008)
D-(+)-glucose	C_6_H_12_O_6_	monosaccharide^28^	0.185 (0.006)
lactose	C_12_H_22_O_11_	disaccharide^28^	0.108 (0.002)
sodium alginate	C_6_H_9_O_7_^–^ Na^+^	marine gelling saccharide^33,35^	0.109 (0.008)
D-alanine	C_3_H_7_O_2_N	amino acid^27^	0.322 (0.012)

a The column compound class shows what the substance could represent in the Arctic aerosol.

b Mass fraction: 55% chloride (Cl^–^), 31% sodium (Na^+^), 8% sulfate (SO_4_^2–^), 4% magnesium (Mg^2+^), 1% potassium (K^+^), 1% calcium (Ca^2+^), and 1% other.

c Likely not following *κ*-Köhler activation due to low solubility^74^, see also Figure S6.

### 3.3 Prediction of the Activation Diameter

**Figure 2:**
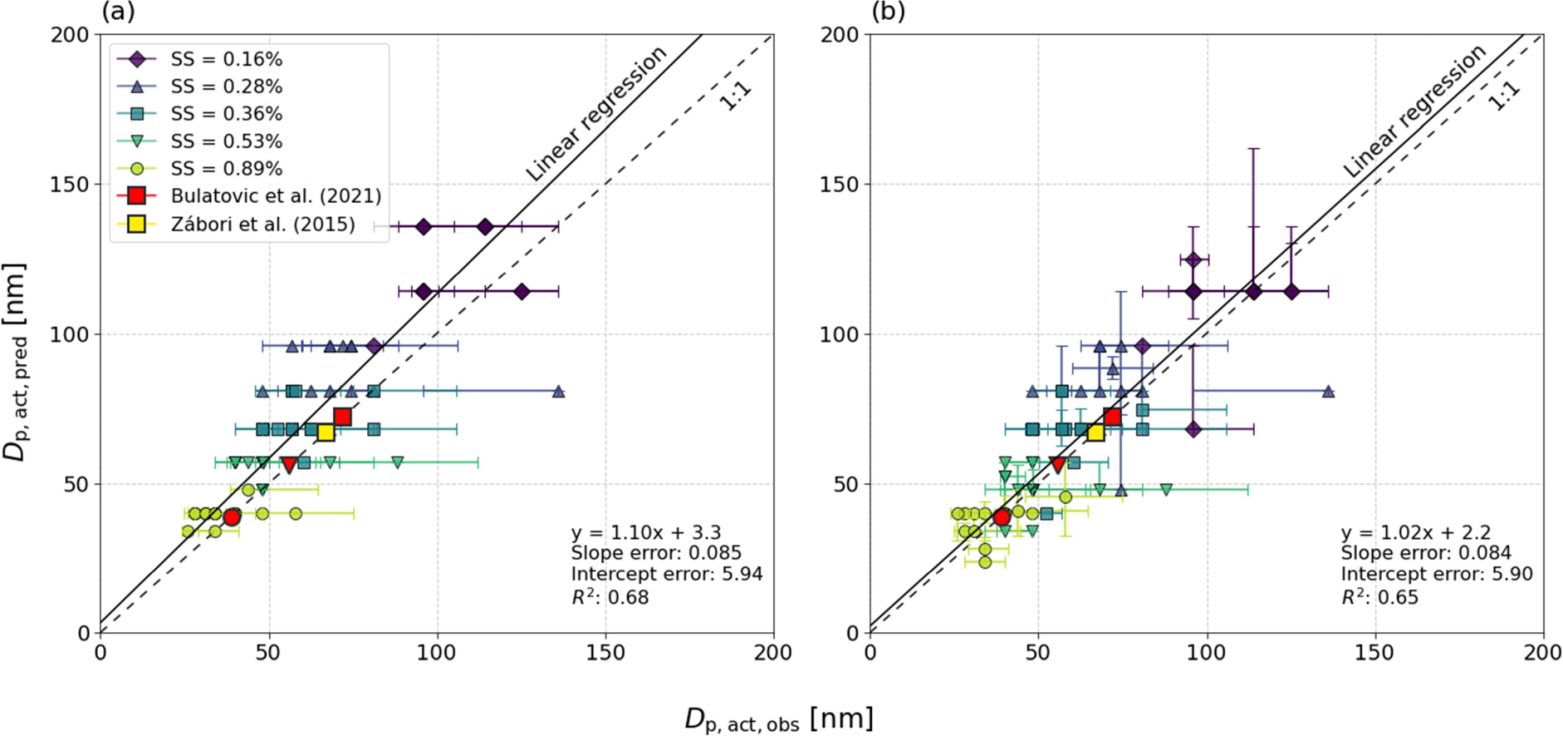
Median (based on FIGAERO-CIMS filter sampling times) CCN activation diameter from *κ*-Köhler calculations (*D*_p,act,pred_) vs field measurements (*D*_p,act,obs_) at different supersaturations (*SS*, 0.16–0.89%). Panel (a) shows the case **FC-tr** (lower time resolution) and panel (b) **AMS-tr** (higher time resolution). The markers at each *SS* level represent one filter sample (**F1**-**F13**) each (filter numbers not shown), and the *SS* level is represented by the marker color and shape. The error bars represent the 25^th^ and 75^th^ percentiles of *D*_p,act_ calculated from CCN number concentrations and size distribution data. The dashed line represents a 1:1 relationship and the solid line the fitted orthogonal linear regression model.

The *κ*_tot,MS_-values in Figure 1b were used for the bottom-up prediction of the activation diameter and later also the CCN number concentration. The relationship between the activation diameters derived from observed CCN (*D*_p,act,obs_) and predictions (*D*_p,act,pred_) is shown for **FC-tr** in Figure 2a and for the high-time-resolution case **AMS-tr** (median of the filter sampling periods) in Figure 2b.

Figure 2 shows that particles in the diameter range of ∼25 to 130 nm activated in the CCNC at *SS* = 0.16–0.89%. The activation diameters of the smallest particles in the Aitken mode (*D*_p_ < ∼70 nm, *SS* = 0.37–0.89%) are in agreement with previous studies from the ASCOS campaign in August 2008 in the High Arctic by Bulatovic *et al*.^79^ and from Ny-Ålesund (Svalbard) in August 2008 by Zábori *et al*..^80^ It is also in this diameter range that the comparison between the median *D*_p,act,obs_ and *D*_p,act,pred_ is closer to the 1:1 line than at lower *SS* (0.16–0.28%), which is mostly evident in the **FC-tr** but also to some degree in the **AMS-tr** case. This shows (*i*) that inclusion of Aitken mode particles is needed at higher *SS* levels to correctly predict the CCN activation diameter, as has been seen earlier for aerosols measured in Svalbard^81^ and (*ii*) that the assumed chemical composition may represent the Aitken mode better than the accumulation mode (*D*_p_ > ∼70 nm, *SS* = 0.16–0.28%) and hence that there could be missing components in the accumulation mode particles which were not successfully incorporated in the analysis. This will be further discussed in Section 3.4.

When fitting an orthogonal linear regression model (not weighted) of the data points at all *SS* levels, the correlation turns out to be fair (*R*^2^ = 0.68 for **FC-tr** and 0.65 for **AMS-tr**). The higher time resolution data in **AMS-tr** resulted in a slope (*m*) closer to 1, a lower intercept (*b*) and similar errors of both *m* and *b* compared to **FC-tr**, as well as a similar *R*^2^. One reason for the better linear fit is likely the larger sample size in **AMS-tr**, but another could be that the chemical composition varied throughout the filter sampling times, which is also evident from Figure 1b. This variation was not captured in **FC-tr** and is also seen as the larger error bars of *D*_p,act,pred_ in Figure 2b compared to Figure 2a. However, the slopes are not statistically different at a 0.05 significance level (*p* = 0.37). The difference between **FC-tr** and the standard case (*κ*_org_ = 0.1) was even smaller (*p* = 0.43 with *m* = 1.01 (error: 0.087), *b* = 6.0 (error: 6.05), *R*^2^ = 0.67). This shows that prediction of *D*_p,act,obs_ by using the molecular composition of the organic fraction from FIGAERO-CIMS did not significantly affect the predictions compared to using the standard *κ*_org_ of 0.1. However, this could be more thoroughly investigated with a higher time resolution of the FIGAERO-CIMS data, which could be achieved by utilizing the full functionality of the FIGAERO inlet with continuous sampling of gas- and particle-phase data *in situ*.

Despite the lack of a significant difference, we chose to continue with **AMS-tr** for the prediction of CCN number concentration because of the higher time resolution. The normalized mean bias (NMB) of the linear regression model shows that the *D*_p,act_ is overpredicted by ∼5% over the whole *D*_p_ range and the normalized mean error (NME) that the uncertainty in the prediction is ∼20%. The regression parameters and uncertainties of both **FC-tr** and **AMS-tr** are shown in Table S7.

### 3.4 Relationship between Supersaturation and Particle Hygroscopicity

**Figure 3:**
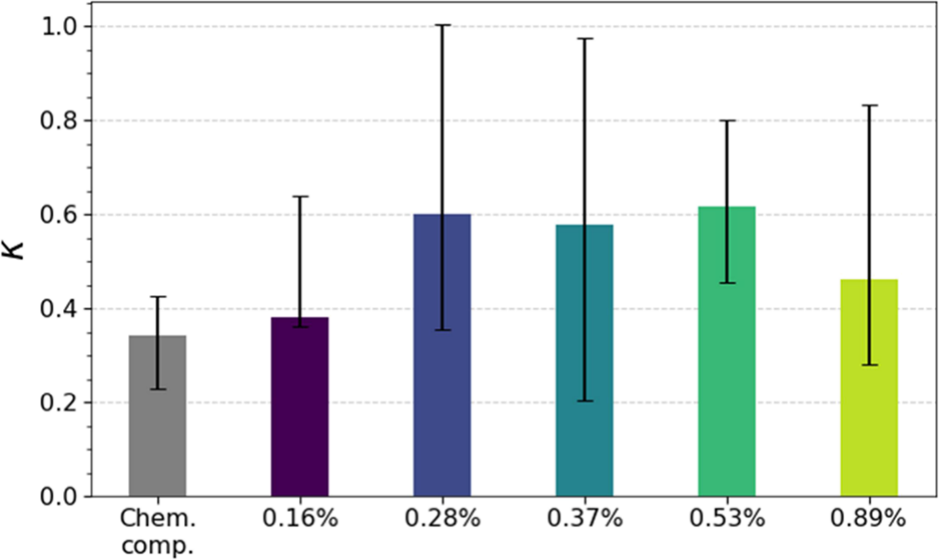
Median hygroscopicity parameter (*κ*) calculated from the chemical composition data (*κ*_tot,MS_, gray bar) and from *D*_p,act,obs_ (*κ*_CCNC_) at different supersaturations (*SS* 0.16–0.89%). Error bars represent the 25^th^ and 75^th^ percentiles.

As mentioned in the previous subsection, *D*_p,act,pred_ appeared to be more similar to *D*_p,act,obs_ at the highest *SS* (0.89%) compared to the lowest *SS* (0.16%). In theory, this could imply that *κ*_tot,MS_ better represents the activation of the smaller particles than the larger particles. To shed some light on this matter, a comparison between the median *κ*_tot,MS_ and *κ*_CCNC_ at the five different *SS* levels is shown in Figure 3. First, all median *κ*_CCNC_-values are higher than the median *κ*_tot,MS_ of 0.37 (mean ± std: 0.39 ± 0.19), which is based on the AMS and FIGAERO-CIMS chemical composition. This is a reflection of the overprediction of *D*_p,act_ in Figure 2, as the lower particle hygroscopicity was counterbalanced by larger diameters. The median *κ*_tot,MS_ value is however only slightly lower than the median *κ*_CCNC_ of 0.38 at *SS* = 0.16%. At *SS* = 0.28–0.53% and 0.89%, the median *κ*_CCNC_ is ∼0.60 and 0.46, respectively, with the 75^th^ percentile reaching up to 0.80–1.0. This indicates that our estimated *κ*_tot,MS_, and hence chemical composition, better represents the larger particles that activate at the lowest *SS* = 0.16% (*D*_p,act_ ≈ 75–125 nm). This is reasonable because the signals of FIGAERO-CIMS and AMS are mass-based, and particles with larger diameters will hence contribute more to the sample signal compared to particles with smaller diameters.

The discrepancies between *κ*_tot,MS_ and *κ*_CCNC_ could have various explanations. A highly hygroscopic inorganic compound not measurable with our mass spectrometers, such as sea salt, could be missing (or an extraordinarily hygroscopic primary organic SSA compound^75^ not represented in our laboratory studies). This hypothesis is supported by findings of sea salt species in the ultrafine aerosol (20–60 nm) during the campaign^29^ but opposed by earlier conclusions from the central Arctic Ocean.^24^ Nonsoluble and surface-active organics, such as fatty acids, could further decrease the surface tension of the growing droplets and hence increase their activation potential.^82^ However, this effect would be most prominent for the smallest particles^83^ and can hence not fully explain the larger variability of *D*_p,act_ at lower *SS* in Figure 2 and slight underprediction of *κ*. Another likely explanation is the broader size bin ranges in the DMPS at larger particle diameters compared to smaller particle diameters, which lead to larger uncertainties in *D*_p,act_. Similarly, we believe that the large variability in *κ*_CCNC_ (Figure 3) could be a matter of sensitivity of the CCN number concentration to variability in the aerosol number size distributions (Figure S7). Depending on where *D*_p,act_ is located, small changes in *D*_p,act_ can largely affect the CCN number, and vice versa, which will result in a larger variability (and possibly larger median offset) in the derived *κ*-values. We conclude that these uncertainties in the instrumental setup are plausible explanations for the variability in deviations between observed and predicted *D*_p,act_ and *κ*-values, but that effects from highly hygroscopic components and organic surfactants cannot be completely ruled out in some cases. This will be further discussed in Section 3.5.

### 3.5 Prediction of CCN Number Concentration

**Figure 4:**
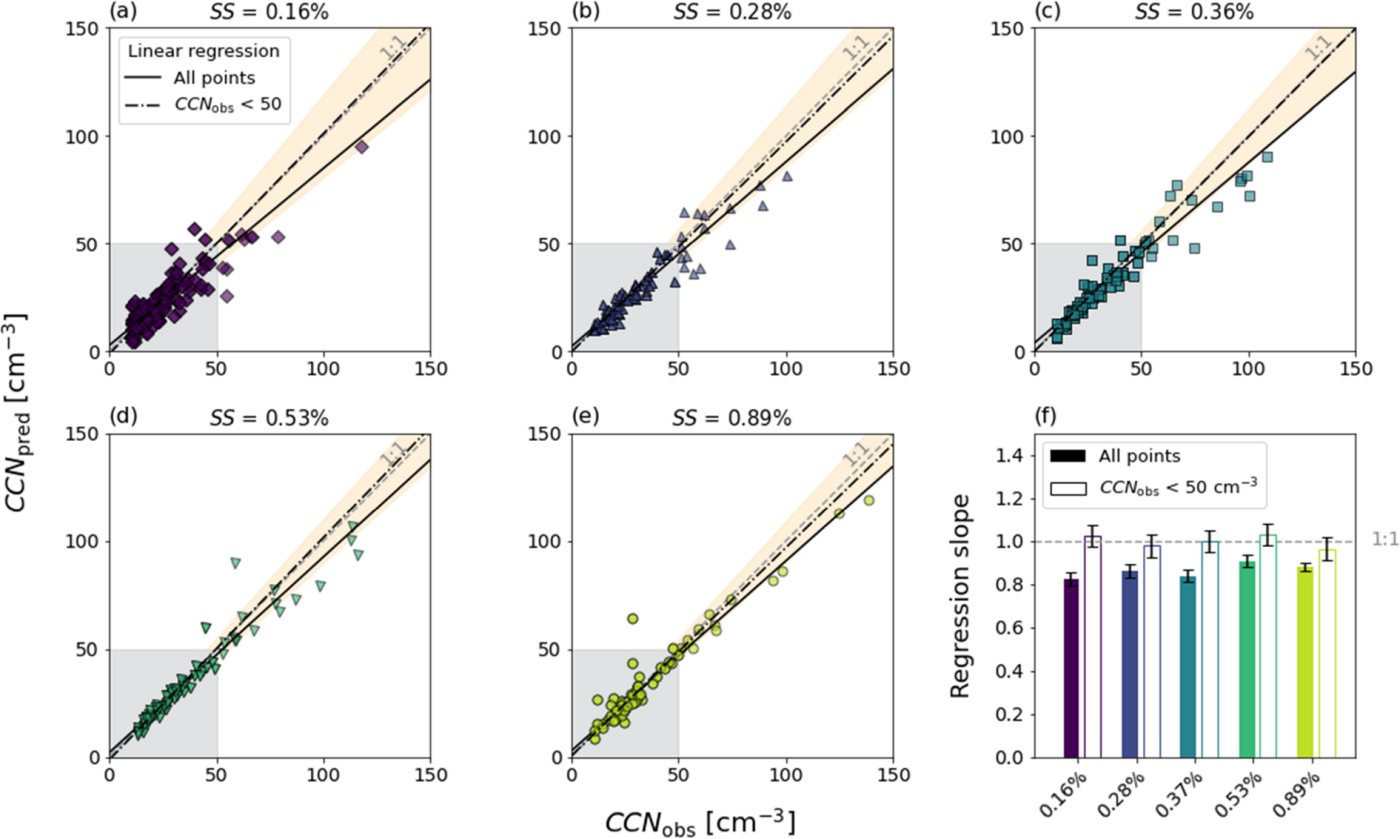
Panel (a–e): Correlation of CCN number concentration from observations (*CCN*_obs_) and calculations with *κ*-Köhler theory (*CCN*_pred_) at different supersaturations (*SS*, 0.16–0.89%). The solid line represents the linear regression model using all data points and the dash-dotted line when only using *CCN*_obs_ < 50 cm^–3^ (shown by the gray-shaded area). The dashed line represents a 1:1 relationship. The NME of the linear regression model is shown as an orange-shaded area. Panel (f) shows the slopes of the linear models with their respective error bars at each *SS* level. Filled bars represent the slopes of all data points and the hollow bars the slopes of *CCN*_obs_ < 50 cm^–3^. The dashed line represents a 1:1 relationship.

The final goal of this CCN closure study is to investigate how well the observed CCN number concentrations can be predicted from chemical composition information and *κ*-Köhler theory. Figure 4 presents the correlation between *CCN*_obs_ and *CCN*_pred_ (calculated using the *κ*-values derived from the chemical composition of the **AMS-tr** case) at the five different *SS* levels using orthogonal linear regression analysis (not weigthed), where the correlation coefficients are listed in Table S8. When fitting the model to all data points per *SS*, the slopes are in the range of 0.82–0.91 (*R*^2^ = 0.82–0.97), whereas when the data are restricted to *CCN*_obs_ < 50 cm^–3^ (76.5–92.5% of the data points), the slopes are increased to 0.96–1.03 (*R*^2^ = 0.67–0.91, which is lower compared to the case with all data points as the variability within the population becomes larger with fewer data points) and the slope error bars at all *SS* levels are encompassing the 1:1 line (Figure 4f). This shows that the high *CCN*_obs_ values are associated with the largest uncertainties, which is reflected in the broad *κ* variations in Figure 3. From Figure 1a, *CCN*_obs_ exceeded 50 cm^–3^ in four samples: **F2-F4** and **F11**. In the case of **F11**, this only occurred at *SS* = 0.16%. In addition to the fact that **F11** was sampled during the transit and hence less stable conditions,^38^ our conclusion is that these high values probably were caused by fluctuations in the instrumentation, possibly because of rapid changes in particle concentration and composition. In **F2-F4**, however, the sampling conditions were more stable with the icebreaker moored and turned upwind. The average wind speed was among the highest during the sampling period (8.2–10.2 m/s) and the wind direction northerly.^38^ It shifted to lower speeds (4.1–8.0 m/s) and easterly direction in sample **F5-F7** when the CCN number concentration was dropping to <50 cm^–3^ again. The aerosol chemical composition analysis (Figure S5) shows that **F2-F4** had a relatively higher SO_4_^2–^-to-Org mass ratio, and the aerosol number size distributions (Figure S7) show that the Aitken-to-accumulation mode ratio was relatively higher compared to the other samples from the ice drift (**F1**, **F5-F7**). Together, these findings point at a different and more hygroscopic aerosol source in **F2-F4** compared to the other samples, and there is a possibility of contributions from, for example, sea salt-sulfate, as opposed to previous results from the High Arctic^24^ but in similarity to findings on submicron SSA in a different region.^84^ The NME of the fit using all data points shows that the uncertainty of the prediction is 9.3–19.1% and that we underestimate *CCN*_obs_ by 4.1–7.6%. This result is considerably better than a previous closure study by Martin *et al*.^16^ for High Arctic aerosols, which showed an overprediction of the CCN number concentration with slopes of 1.09–1.44 (for the case closest to ours with *κ*_org_ = 0.1, *κ*_sulfate_ = 0.7, *ρ*_org_ = 1.2 g cm^–3^, and a soluble organic fraction). Our organic fraction had a mean *κ*_org_ of 0.08 (std: 0.04) and behaved in the *κ*-Köhler model as fully soluble, whereas they concluded that *κ*_org_ has to be as low as 0.02, in practice meaning a “sparingly soluble to effectively insoluble” organic fraction.^16^ Because we measured a considerably higher organic mass fraction of 60% (std: 28%) compared to their 36% and our organic fraction was more hygroscopic, the organic fraction contributed much more to the total *κ*-value in our study.

We show with our closure study that the inorganic fraction of the submicron aerosol in the High Arctic late summer can in general be chemically explained as acidic and therefore highly hygroscopic. This is comparable to other remote marine environments (*e.g.*, South Atlantic,^85^ sub-Arctic northeast Pacific Ocean,^86^ and Southern Ocean^87^) where the aerosol is locally produced and big ammonium sources such as sea bird colonies^88^ are lacking. Our results (using all data points in Figure 4) show that the *κ* would even need to be larger rather than smaller, that is, the aerosol particles be more hygroscopic, to fully match the observed *D*_p,act_ and CCN concentrations. This further justifies the exclusion of ammonium species in our calculations.

We further show that the cloud droplet activation potential of High Arctic summertime aerosols can be generally explained by the common *κ*-Köhler theory, where the organic compounds behave as fully soluble in water. However, clouds in the summertime Arctic are normally mixed-phase^89^ (consisting of both water droplets and ice), which was also the case during most of our expedition.^45^ Measured INP^48^ concentrations between September 11 and September 19 were however relatively low and exhibited a low ice-nucleating ability (freezing temperature at 0.1 INP L^–1^ was around −25 to −30 °C). This indicates that the clouds were largely composed of liquid droplets and that *κ*-Köhler theory can be used to predict the cloud activation, which simplifies the description of the aerosol–cloud interactions.

The measured aerosol chemical composition and *κ*-values derived from the field samples and the laboratory experiments show that the organic compounds are marine (primary and secondary) in nature.^9^ In a continuously warming climate, aerosol emissions are expected to change because of a decreased sea ice extent during summer, changes in the algal communities and biogeochemical cycles,^90^ more frequent wildfires around the Arctic Ocean,^91^ advected pollution from mid-latitudes,^92^ and influence from ship emissions.^93^ More open water in contact with the atmosphere combined with increased traffic will lead to larger amounts of aerosol particles in this currently CCN-limited regime. If this will result in cloud brightening, as has been seen in other marine regions,^94^ or in counter-effects by increased cloud glaciation, as predicted by models,^95^ remains to be observed.

## Supporting Information Available

The document includes additional experimental details, materials and methods, and further statistical details of the results. (PDF)
