# Supplementary material for: Using Novel Molecular-Level Chemical Composition Observations of High Arctic Organic Aerosol for Predictions of Cloud Condensation Nuclei
Source: Environ Sci Technol. 2022 Sep 16;56(19):13888–99. doi: 10.1021/acs.est.2c02162 (PMC9535938; doi:10.1021/acs.est.2c02162)
Supplement: Supplementary file 1 — es2c02162_si_001.pdf [file es2c02162_si_001.pdf]

Supporting Information for:

Using novel molecular-level chemical composition observations of High Arctic organic aerosol for predictions of cloud condensation nuclei

Karolina Siegel[1,2,3], Almuth Neuberger[1,3], Linn Karlsson[1,3], Paul Zieger[1,3], Fredrik Mattsson[1,3], Patrick Duplessis[4], Lubna Dada[5], Kaspar Daellenbach[5], Julia Schmale[6], Andrea Baccarini[6], Radovan Krejci[1,3], Birgitta Svenningsson[7], Rachel Chang[4], Annica M.L. Ekman[2,3], Ilona Riipinen[1,3], and Claudia Mohr[1,3,*]

[1] Department of Environmental Science, Stockholm University, Stockholm, SE-10691, Sweden.

[2] Department of Meteorology, Stockholm University, Stockholm, SE-10691, Sweden.

[3] Bolin Centre for Climate Research, Stockholm University, Stockholm, SE-10691, Sweden.

[4] Department of Physics and Atmospheric Science, Dalhousie University, Halifax, CA-B3H 4R2, Canada

[5] Laboratory of Atmospheric Chemistry, Paul Scherrer Institute, Villigen, CH-5232, Switzerland

[6] Extreme Environments Research Laboratory, École Polytechnique Fédérale de Lausanne, Sion, CH-1951, Switzerland.

[7] Division of Nuclear Physics, Lund University, Lund, SE-22100, Sweden.

[*] Corresponding author: Claudia Mohr, claudia.mohr@aces.su.se

Supporting Information Contents

S1 Instrumentation and sampling details..3

S2 Laboratory experiments on CCN activation potential..5

S3 Assumptions made for predictions using κ-Köhler theory.....................................12

S3.1 FIGAERO-CIMS and AMS comparison...12

S3.2 Origin of inorganic aerosol components and black carbon.....................................15

S4 Predictions of activation diameter and CCN number concentration.......................19

S4.1 Constraints applied to the dataset...20

References 26

S1 Instrumentation and sampling details

The Aerosol Mass Spectrometer (AMS, Aerodyne Research Inc., USA) was collecting data every minute and was alternating measurements between a whole-air and a PM_1 (particulate matter < 1 µm in aerodynamic diameter) inlet. The particle number size distributions in the diameter (D_p) range of 10 nm - 9.7 µm, used for calculations of total particle number concentration, were measured by a differential mobility particle sizer (DMPS), consisting of a custom-built differential mobility analyser (DMA) in connection to a mixing condensation particle counter (MCPC, Brechtel Manufacturing Inc., Model 1720, USA) and a WELAS 2300HP aerosol spectrometer with a Promo 2000H system (Palas GmbH, Germany)[1,2]. One full-size distribution scan took 9 min. The DMPS and WELAS number size distributions were combined at a diameter of 300 nm, as the DMPS size distribution is more likely to be influenced by multiply charged particles above that size and the WELAS tends to undercount particles smaller than ~300 nm. The DMPS and WELAS data were corrected for losses through diffusion, impaction and sedimentation using the Particle Loss Calculator by Von der Weiden et al.[3] For the loss calculations, a common particle density of 1.30 g cm^{-3} was calculated based on average contributions of organics (Org) and non-sea-salt sulfate in the AMS data for submicron particles and used for the entire diameter range (0.01-10 µm)[4].

The cloud condensation nuclei counter (CCNC, Droplet Measurement Technologies, USA, Model CCN-100) measured the number of ambient aerosol particles that activated into cloud droplets at a known supersaturation and recorded cloud droplet size distributions in the range of 0.75-10 µm every second. It was sampling behind the whole-air inlet in parallel to the FIGAERO-CIMS filter sample collection at a sample flow of 50 cc min^{-1}, and a sheath flow of 450 cc min^{-1} for a total flow of 500 cc min^{-1}. The CCNC was calibrated using size-resolved ammonium sulfate particles with an assumed κ-value of 0.6 for five different supersaturation settings with the method described in Rose et al.[5] Based on the

calibrations, these supersaturation settings were calculated to be 0.16, 0.28, 0.37, 0.53, and 0.89%. During ambient measurements, the instrument cycled through the five supersaturation settings, in descending order, 20 minutes at the first setting and 10 minutes at each of the other four, for a total time of 1 hour for one full cycle. For the analysis, the 9-minute median values were used corresponding to the aerosol size distribution scanning time of the combined DMPS/WELAS system.

S2 Laboratory experiments on CCN activation potential

Sea salt and seven organic compounds (see Table S1) were dissolved individually in MilliQ® water to concentrations of 0.01-0.08 M (undecanoic acid as a suspension, which was achieved with an ultrasonic bath for 20 min, due to the low solubility in water). A nebuliser (MEDIX, Clement Clarke, UK) with a compressed air flow was used to produce aerosol particles from the solutions. The air stream was dried to RH < 10% (measured by a Rotronic RH sensor, model HC2-C04) in a silica gel-based diffusion drier (L: 0.8 m, D: 0.06 m) before entering the differential mobility analyser (DMA, Custom made short Vienna-type, sample flow 1.47 lpm, sheath air flow 9.2 lpm) for particle size selection. The sample air was thereafter divided, with 0.97 lpm going to a condensation particle counter (CPC, TSI, No. 3010), and 0.50 lpm to a CCNC (Droplet Measurement Technologies, USA, Model CCN-100). The instruments were connected by stainless steel tubing (i.d. 2 mm) and short pieces of conductive silicone tubing (i.d. 3 and 7 mm, total length ~1 m).

The DMA was calibrated with polystyrene latex (PSL) spheres (Thermo Fischer Scientific, USA) of 100 ± 3 nm and 203 ± 5 nm diameter (see Figure S1). The CCNC was calibrated with ammonium sulfate ($(NH_4)_2SO_4$, VWR) according to the method described in Rose et al. (2008)[5]. The result of the calibration is shown in Figure S2. The calibration was validated with sodium chloride (NaCl, Sigma Aldrich), where the experimentally derived activation diameter of NaCl at different supersaturation ratios (*SS*) was compared to those derived from the UManSysProp model (v1.0)[6] of CCN activation potential (Figure S3).

The activation diameter ($D_{p,act}$) of sea salt was determined at *SS* 0.2-0.35% and of the organic substances at *SS* 0.2-1%. The smaller SS range for sea salt was due to the much higher hygroscopicity of sea salt ($\kappa \approx 1.1$)[7] compared to oxygen-containing organics ($\kappa \leq 0.3$).[8] The dry diameter scanning range in the DMA was 30-200 nm (20 size bins) for both sea salt and organic solutions. For each *SS*, 5 scans were performed with a residence time of 15s per

size bin and a lag time of 5s in-between size bins. The $D_{p,act}$/SS pairs were then used with Eq. (3) in the main manuscript to calculate hygroscopicity parameters (κ) for the different compounds.

Table S1. The different substances used for the experimental determination of CCN activation potential.

Compound	Molecular formula	Manufacturer	Purity
Sea salt	Mix of inorg. ions*	Sigma Aldrich	99%
Levulinic acid	$C_5H_8O_3$	Sigma Aldrich	For synthesis
Succinic acid	$C_4H_6O_4$	Sigma Aldrich	≥99.0%
Undecanoic acid	$C_{11}H_{22}O_2$	Sigma Aldrich	For synthesis
D-(+)-glucose	$C_6H_{12}O_6$	Sigma Aldrich	99.5%
Lactose	$C_{12}H_{22}O_{11}$	Merck	For biochemistry
Sodium alginate	$C_6H_9O_7^- Na^+$	Fisher scientific	100%
D-alanine	$C_3H_7O_2N$	Alfa Aesar	99%

* Artificial sea salt mixture, Sigma Aldrich S9883; mass fraction: 55% chloride (Cl^-), 31% sodium (Na^+), 8% sulfate (SO_4^{2-}), 4% magnesium (Mg^{2+}), 1% potassium (K^+), 1% calcium (Ca^{2+}) and 1% other

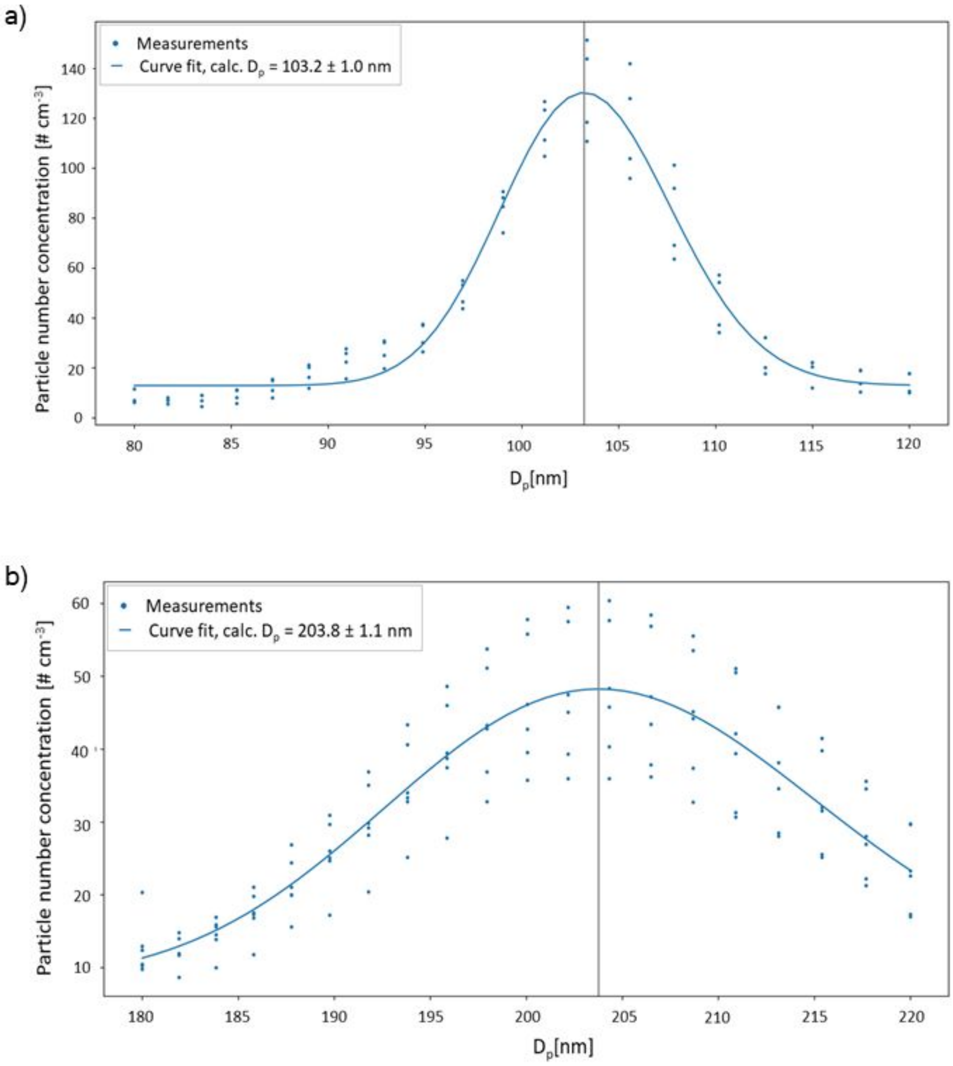

Figure S1. DMPS calibrations with polystyrene latex (PSL) spheres (Thermo Fischer Scientific, USA) of a) 100 ± 3 nm and b) 203 ± 5 nm diameter. The points represent individual measurements of a total of 5 D_p scans, and the solid curve the best fit to the points. The vertical line shows the maximum D_p of the curve fit (written out in the legend ± 1 standard deviation of the mean).

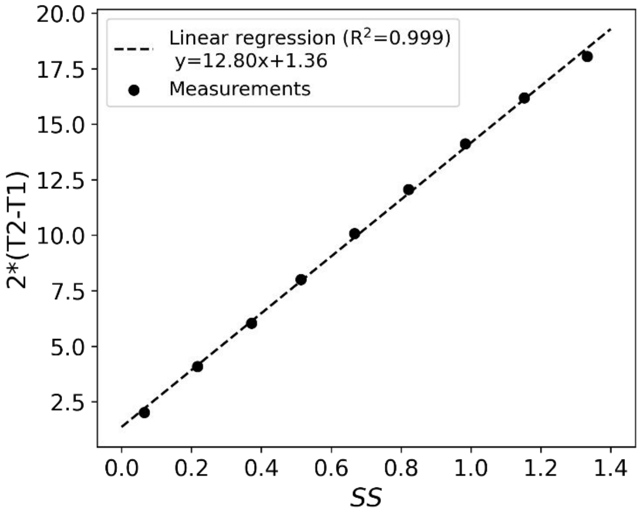

Figure S2. Calibration curve of the CCNC (using ammonium sulfate, $(NH_4)_2SO_4$) as described by the method in Rose et al., (2008)[5].

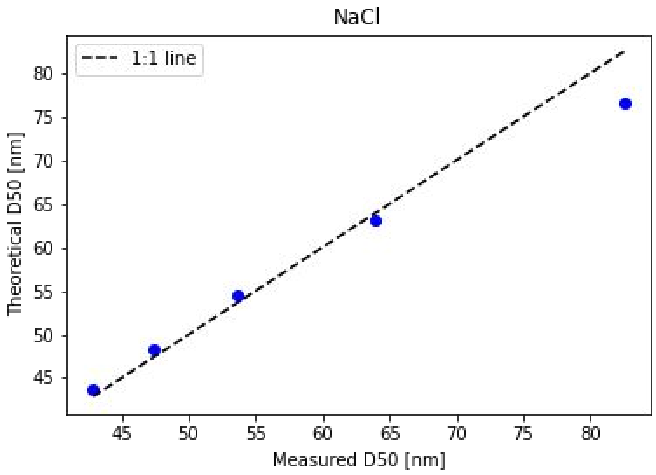

Figure S3. Validation of the CCNC calibration by comparison of measured D50 (activation diameter) of sodium chloride (NaCl) to theoretical D50.[6]

Table S2. Median signal- and mass-weighted M_s of each filter sample as analysed by FIGAERO-CIMS, AMS and MAAP. The κ_{org} values were calculated from Eq. (1) in the main manuscript. These values were used as input for the organic aerosol fraction in the κ-Köhler calculations of the **FC-tr** case.

**Filter number**	M_{org} **[g mol^-1]**	κ_{org}	M_{tot} **[g mol^-1]**	κ_{tot}
F1	204.5	0.11	179.7	0.25
F2	187.4	0.12	157.3	0.32
F3	193.2	0.12	156.3	0.34
F4	173.2	0.13	146.4	0.33
F5	174.3	0.13	143.4	0.36
F7	154.2	0.14	142.7	0.26
F8	172.9	0.13	144.7	0.26
F9	155.4	0.14	141.8	0.28
F10	191.5	0.12	154.7	0.35
F11	168.0	0.13	158.6	0.21
F12	163.3	0.14	130.4	0.42
F13	180.1	0.13	122.3	0.53

S3 Assumptions made for predictions using *κ*-Köhler theory

S3.1 FIGAERO-CIMS and AMS comparison

When using the FIGAERO-CIMS data to represent the organic mass fraction in its entirety, it is assumed that Org measured by AMS consists of only semi-volatile and oxygenated compounds (i.e. the compounds the iodide-FIGAERO-CIMS is sensitive to). This is a simplification, as the ionization technique of the AMS (electron impact) is less selective and able to also detect e.g. non-oxygenated hydrocarbons. In addition, aerosol particles in the AMS are flash-vaporized at 600°C, whereas the maximum desorption temperature in the FIGAERO-CIMS is 200°C. A comparison between the measured AMS Org mass and the mass of organic compounds measured by FIGAERO-CIMS (using a previously reported maximum sensitivity of the instrument at the collisional limit to estimate atmospheric concentrations)[9] shows that the FIGAERO-CIMS on average measured 75% (standard deviation (std) 77%) of the AMS Org mass, comparable to other locations where the two instruments were running in parallel[10–12]. Because of pristine conditions and low aerosol mass loadings in the summertime High Arctic atmosphere, the measured AMS concentrations were close to the detection limit[13] (mean concentration during filter deposition periods: 53 ng m^{-3}), and the standard deviation is very high. However, the comparison indicates that a large fraction of the organic aerosol in the central Arctic Ocean in late summer is oxygenated. A calculation of the mass-weighted oxygen-to-carbon (O:C) and hydrogen-to-carbon (H:C) ratios of the two instruments can give an additional estimate of how well the AMS Org fraction is represented by the FIGAERO-CIMS organics. The mean O:C value of the filter samples was 0.90 (std: 0.33) for the AMS and 0.59 (0.11) for the FIGAERO-CIMS, and the median H:C value 1.61 (0.19) for the AMS and 1.63 (0.06) for the FIGAERO-CIMS (see Table S3 for values of the individual samples). This shows that on average the AMS measured compounds with a similar carbon chain length, but a somewhat higher degree of oxygenation compared to the FIGAERO-CIMS. This is likely due to compounds that only evaporate at higher temperatures than the 200°C of the FIGAERO-

CIMS. We suggest that one important compound class that is detected by the AMS but missing in the FIGAERO-CIMS is saccharides. Saccharides have been found to be present in High Arctic aerosol[14] and commonly have an O:C ratio close to 1, but they were not found in the FIGAERO-CIMS mass spectrum. It is also likely that the AMS detects other primary organic compounds that would not evaporate at the 200°C of the FIGAERO-CIMS.

Table S3. Oxygen-to-carbon (O:C) ratios and hydrogen-to-carbon (H:C) ratios of the AMS organic (Org) fraction and FIGAERO-CIMS organic (*CHO*, *CHON*, *CHONS*, *CHOS*) classes. Filter numbers refer to FIGAERO-CIMS samples and the AMS data was hence averaged to the same start and end times.

	O:C ratio		H:C ratio	
**Filter number**	**AMS**	**FIGAERO-CIMS**	**AMS**	**FIGAERO-CIMS**
F1	1.09	0.48	1.37	1.56
F2	0.97	0.65	1.41	1.57
F3	1.02	0.50	1.41	1.70
F4	1.33	0.48	1.45	1.63
F5	1.20	0.54	1.57	1.58
F7	0.89	0.60	1.68	1.61
F8	0.18	0.65	1.94	1.58
F9	0.59	0.53	1.89	1.65
F10	0.81	0.46	1.65	1.73
F11	0.42	0.78	1.89	1.72
F12	1.10	0.62	1.52	1.59
F13	1.22	0.78	1.54	1.63
Mean	0.90	0.59	1.61	1.63
Standard deviation	0.33	0.11	0.19	0.06

S3.2 Origin of inorganic aerosol components and black carbon

For the predictions presented in this study, SO_4^{2-} was assumed to have contributions from sulfuric acid (H_2SO_4, $\kappa \sim 0.7$)[15] instead of ammonium sulfate ($(NH_4)_2SO_4$, $\kappa = 0.61$)[16], due to the insignificant amount of NH_4^+ in the AMS data. Further, sea-salt sulfate has previously been shown to have a negligible contribution to the submicron sulfate mass[17]. Cl^- and NO_3^- were similarly hypothesized to be mainly present as acids, i.e. hydrochloric acid (HCl, $\kappa = 1.42$ based on Eq. (1) in the main manuscript) and nitric acid (HNO_3, $\kappa = 0.85$ based on Eq. (1)) instead of ammonium chloride (NH_4Cl, $\kappa = 1.03$ based on Eq. (1)) and ammonium nitrate (NH_4NO_3, $\kappa = 0.67$)[16], respectively. Due to the high hygroscopicity of the aerosol, the particles likely have a high water content, where the water can act as a base in the absence of NH_4^+.

This assumption represents an upper limit of the aerosol hygroscopicity as measured by the AMS, and other estimations could be valid as well. To test this, we did two sensitivity runs: 1) SO_4^{2-} was assumed to consist of 50% H_2SO_4 and 50% MSA ($\kappa = 0.32$)[18], the NO_3^- attributed to organonitrates (which made up 1-7% of the FIGAERO-CIMS filter samples) and the small amounts of Cl^- neglected. The orthogonal linear regression correlation between $D_{p,act,obs}$ and $D_{p,act,pred}$ had then the equation y = 1.19x + 3.8. 2) Applying a heterogeneous chemical composition throughout the size distribution, using a different composition of the Aitken mode ($D_p < 60$ nm) particles[19] with H_2SO_4 as SO_4^{2-}, ammonium bisulfate (as concluded by the authors of the study) as NH_4^+, sea salt[7] as sodium, the organic κ per filter sample from FIGAERO-CIMS for SOA, and the κ (0.11) of lactose and sodium alginate from the laboratory experiments for polysaccharides. The orthogonal linear regression equation of $D_{p,act,obs}$ vs $D_{p,act,pred}$ for this case was y = 1.02x + 2.2, which is exactly the same as for the **AMS-tr** case. Hence, the use of this heterogeneous chemical composition did not affect the predictions, which is in line with earlier studies[20].

Black carbon (BC) levels are normally very low in the pristine Arctic summer

atmosphere[21,22], and the concentrations of equivalent BC (eBC) were higher when *Oden* was in transit compared to when moored to the ice. Sampled eBC was thus assumed to originate from an undesired sampling of ship stack from *Oden* and therefore fresh and non-aged. From this, it was assumed to mainly consist of pure carbon (C) and therefore to be completely non-hygroscopic with a κ value of 0.[23]

Table S4. Example of how the $\kappa_{tot,MS}$ were calculated for the two cases **FC-tr** and **AMS-tr**, showing filter samples **F1** and **F13**. In the case with lower time resolution (**FC-tr**), the median mass per FIGAERO-CIMS filter sample of each AMS species was used to calculate $\kappa_{tot,MS}$. In the higher time resolution case (**AMS-tr**), one $\kappa_{tot,MS}$ was calculated for each time step, and a median value per filter sample was thereafter calculated as comparison to **FC-tr**.

FC-tr

Date and time	Filter number	Median m_{Org}	Median m_{SO4}	Median m_{NO3}	Median m_{NH4}	Median m_{Cl}	Median m_{BC}	Median $\kappa_{tot,MS}$
2018-09-11 18:42:00	F1	0.05279	0.013423	0.0007329	0.000000	0.0008433	0.000000	0.25
…	…	…	…	…	…	…	…	…
2018-09-12 03:37:30	F13	0.08564	0.04062	0.0008795	0.000000	0.0009787	0.000000	0.53

AMS-tr

Date and time	Filter number	m_{Org}	m_{SO4}	m_{NO3}	m_{NH4}	m_{Cl}	m_{BC}	$\kappa_{tot,MS}$	Median $\kappa_{tot,MS}$
2018-09-11 14:04:00	F1	0.036749	0.008977	0.001673	0.000129	0.002803	0.000000	0.31	
…	…	…	…	…	…	…	…	…	0.29
2018-09-11 23:13:00	F1	0.110911	0.052953	0.000693	0.000000	0.002118	0.010000	0.30	
2018-09-19 14:40:00	F13	0.000000	0.006557	0.000000	0.000000	0.000000	0.000000	0.70	
…	…	…	…	…	…	…	…	…	0.63
2018-09-19 23:31:00	F13	0.008751	0.1571	0.000000	0.00005590	0.000000	0.000000	0.67	

Table S5. Values found in the literature of the molecular weight (M_s), density (ρ_s) and κ for the inorganic aerosol fraction, used as input in the κ-Köhler calculations.

**Compound**	**Assumed origin**	M_s **[g mol^{-1}]**	ρ **[g cm^{-3}]**	κ
SO_4^{2-}	H_2SO_4	98.08	1.83	0.70 [ref. 15]
NO_3^-	HNO_3	63.01	1.48	0.85*
Cl^-	HCl	35.45	1.40	1.42*
BC	Non-aged ship stack	12.01	1.80	0.00 [ref. 23]

* Calculated from Eq. (1) in the main manuscript

S4 Predictions of activation diameter and CCN number concentration

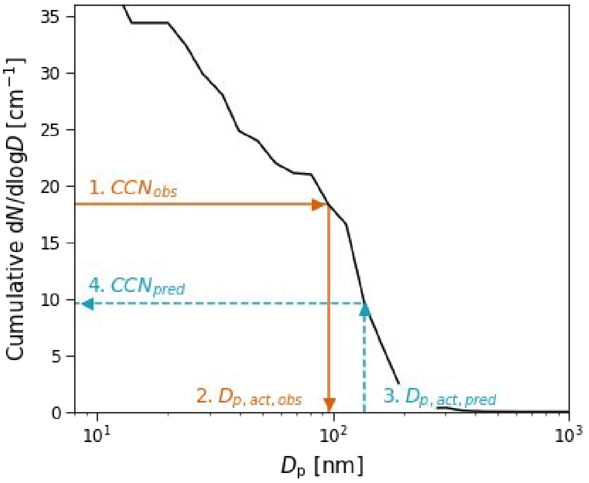

Figure S4. Example of the procedure for calculation of the observed activation diameter ($D_{\mathrm{p,act,obs}}$) and the bottom-up prediction of the CCN number concentration (CCN_{pred}). The observed CCN number concentration (CCN_{obs}) is known (1) and $D_{\mathrm{p,act,obs}}$ can be estimated by matching CCN_{obs} with the cumulative number size distribution data, starting from the largest diameter (2). The predicted $D_{\mathrm{p,act,pred}}$ is calculated based on the $\kappa_{\mathrm{tot,MS}}$ derived from the chemical composition data (3), which is matched with the size bins of the number size distribution data to find CCN_{pred} (4).

S4.1 Constraints applied to the dataset

For further analysis of the CCN dataset, we set some constraints on the dataset for what was considered to be useful data. Time points with CCN concentrations < 10 cm^{-3} were removed due to high fluctuations in $D_{p,act,obs}$ up to 10 µm, which were thought to be caused by uncertainties close to the detection limit of the CCNC. Rows with κ_{CCNC} values > 2 were also considered unreasonable and hence removed, while at the same time providing a lower limit for $D_{p,act,obs}$ at each *SS* setting. For the analysis of CCN number prediction, outliers in the ratio CCN_{pred}/CCN_{obs} were detected as 3 standard deviations from the mean, and the corresponding CCN_{pred} and CCN_{obs} values were replaced by their respective medians. Outliers were removed as they were shown to have a large effect on the final correlation coefficients in many cases. The correlation between $D_{p,act,obs}$ and $D_{p,act,pred}$, as well as between CCN_{obs} and CCN_{pred}, then shows how well κ-Köhler theory with the measured aerosol chemical composition as input predicts the observations.

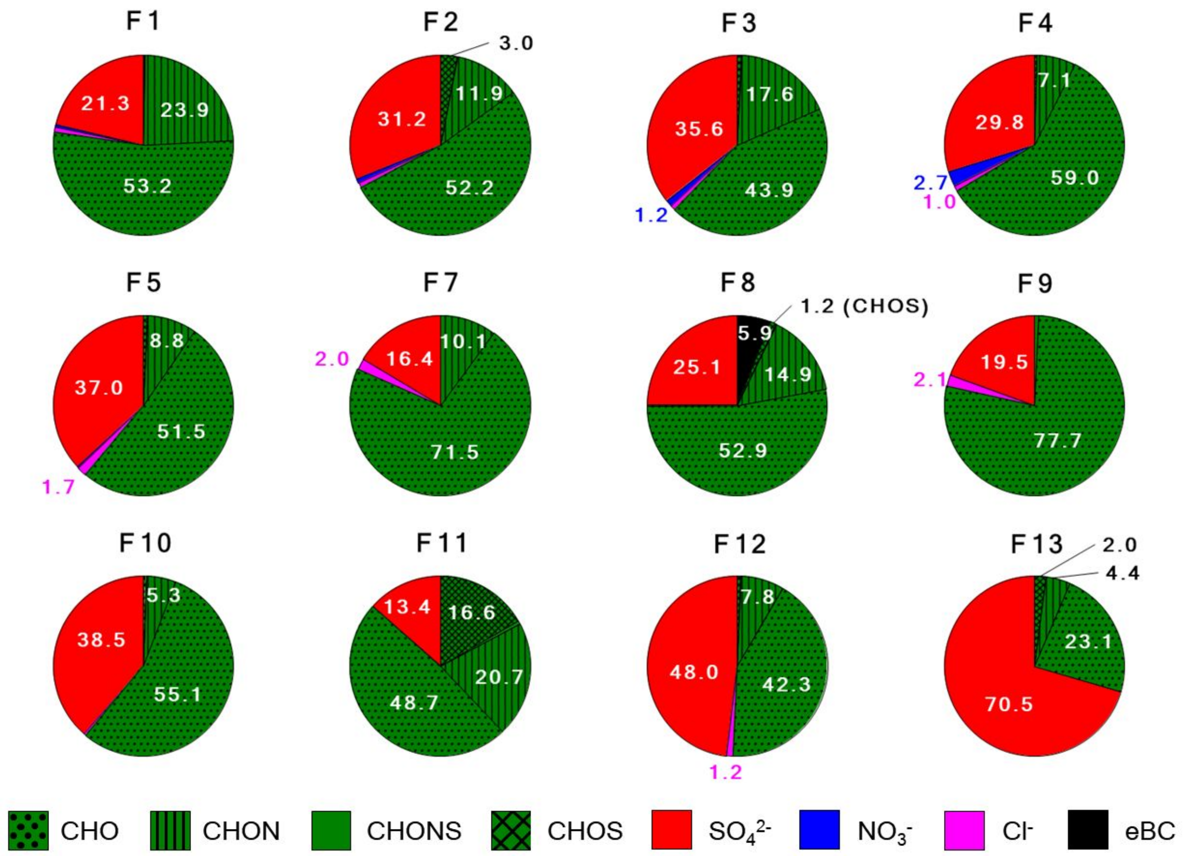

Figure S5. Pie charts of the median chemical composition (Org, inorganic and eBC) of each filter sample (filter number shown above each chart), where the Org is divided into FIGAERO-CIMS compound categories (*CHO*, *CHON*, *CHONS* and *CHOS*, meaning molecules containing carbon, hydrogen, oxygen + nitrogen and/or sulfur), scaled to total Org. Percentages are given as numbers either inside or outside of the charts (numbers < 1% are not shown).

Table S6. Median mass contribution [%] of the inorganic species (sulfate, nitrate, ammonium, chloride) and organics (all measured by AMS) and elemental black carbon (eBC) to each of the FIGAERO-CIMS filter samples.

**Filter number**	**SO_4^{2-}** [%]	**NO_3^-** [%]	**NH_4^+** [%]	**Cl^-** [%]	**Org** [%]	**eBC** [%]
F1	21.28	0.536	0.000	0.839	77.34	0.000
F2	31.15	0.788	0.000	0.880	67.18	0.000
F3	35.60	1.22	0.000	0.912	62.26	0.000
F4	29.82	2.68	0.000	1.04	66.46	0.000
F5	36.95	0.192	0.000	1.74	61.12	0.000
F7	16.37	0.000	0.000	2.01	81.62	0.000
F8	25.10	0.000	0.000	0.000	69.03	5.87
F9	19.46	0.000	0.000	2.06	78.48	0.000
F10	38.54	0.000	0.000	0.494	60.97	0.000
F11	13.40	0.000	0.000	0.000	86.60	0.000
F12	48.04	0.000	0.000	1.21	50.74	0.000
F13	70.50	0.000	0.000	0.000	29.50	0.000

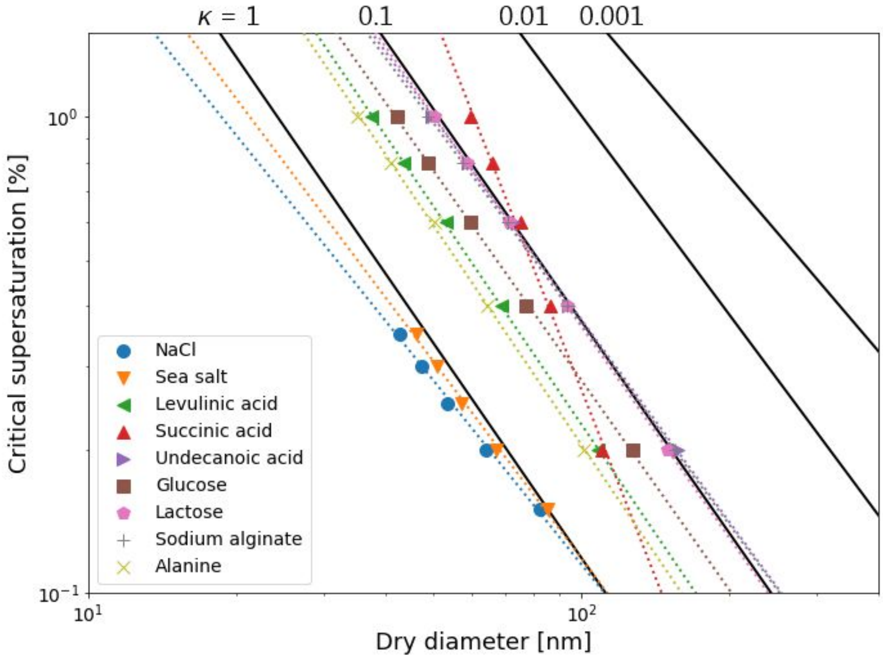

Figure S6. Critical supersaturation vs dry activation diameter (D50 in Fig. S3) of the compounds used in the laboratory experiments. NaCl was used as a validation of the setup and comparison to the other substances, which was shown to have a κ value of 1.4. This is in line with the value presented by Petters & Kreidenweis (2007)[16], which was later updated to 1.5 by Zieger et al. (2017)[7]. Individual data points are shown as markers and linear regression lines as dashed lines. Black solid lines represent calculated critical supersaturation for $0.001 \leq \kappa \leq 1$ using $T = 298.15$ K and $\sigma = 72.8$ mN m^{-1}. Due to low solubility, the activation of succinic acid was likely not following κ-Köhler theory and the curve has therefore a different slope compared to the other substances[24].

Table S7. Slope (*m*), intercept (*b*) and correlation coefficient R^2 of the orthogonal linear correlation of the observed ($D_{p,act,obs}$) vs predicted ($D_{p,act,pred}$) activation diameters over the whole *SS* range (0.16-0.89%) for the cases **FC-tr** (Fig. 2a in the main manuscript) and **AMS-tr** (Fig. 2b). *N* is the number of data points used for the regression analysis, NMB is the normalized mean bias and NME the normalized mean error.

**Case**	***N***	***m***	***b***	R^2	**NMB [%]**	**NME [%]**
**FC-tr**	60	1.10	3.3	0.68	15.0	23.3
**AMS-tr**	60	1.02	2.2	0.65	5.19	19.9

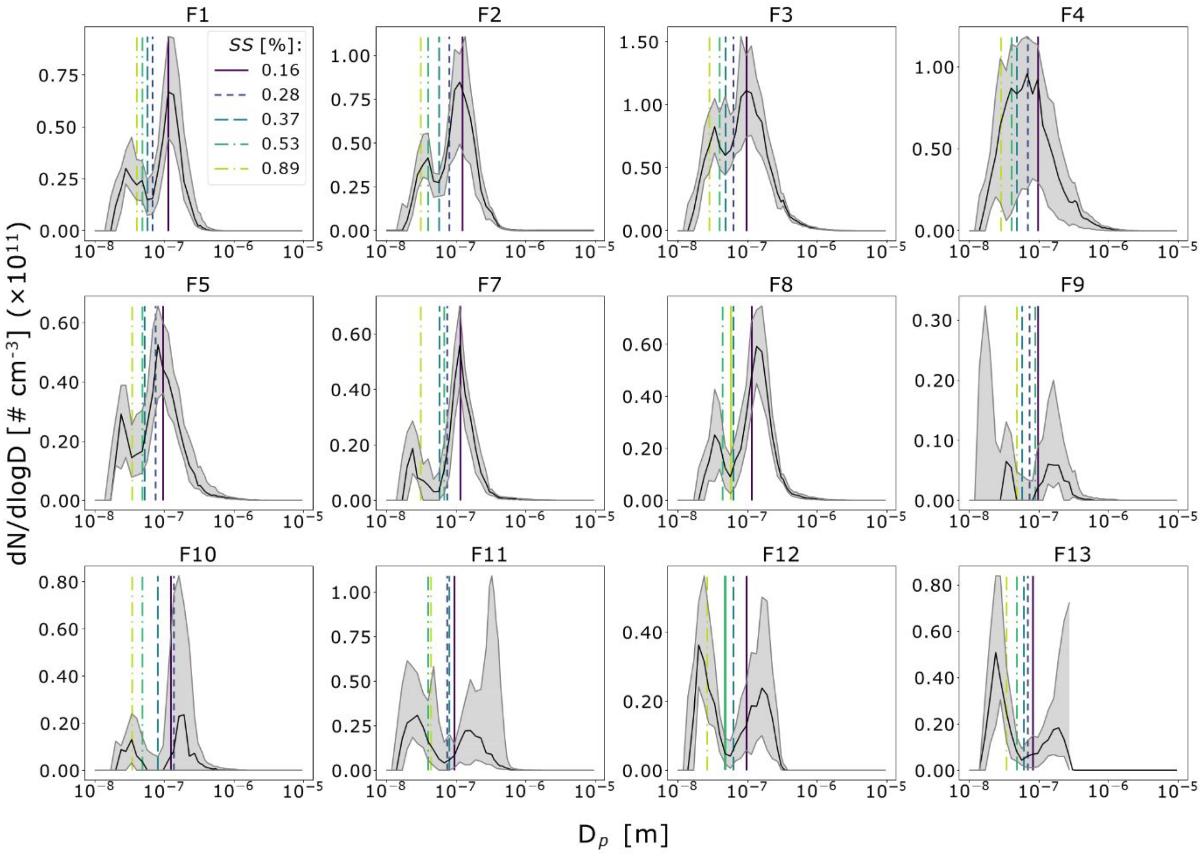

Figure S7. Median number size distributions of each filter sample (**F1**-**F13**). The shaded area represents the values within the 25th and 75th percentiles, and the vertical lines represent the observed activation diameter ($D_{p,act,obs}$), i.e. the smallest activated particle, at different supersaturations (*SS*).

Table S8. Slope (*m*), intercept (*b*) and correlation coefficient R^2 of the orthogonal linear correlation of the observed (CCN_{obs}) vs predicted (CCN_{pred}) CCN number concentration per *SS* level (shown in Fig. 4 in the main manuscript). *N* is the number of data points used for the regression analysis, NMB is the normalized mean bias and NME the normalized mean error. The table is divided into an upper part for the regression models using all data points and a lower part for the models using only CCN_{obs} < 50 cm^{-3}.

All CCN_{obs} points						
***SS* [%]**	***N***	***m***	***b***	R^2	**NMB [%]**	**NME [%]**
0.16	158	0.82	2.8	0.82	-7.2	19.1
0.28	81	0.86	2.2	0.91	-7.6	14.2
0.37	88	0.84	3.8	0.92	-5.6	12.6
0.53	72	0.91	2.0	0.94	-4.3	10.2
0.89	70	0.88	2.9	0.97	-4.1	9.3
CCN_{obs} < 50 cm^{-3}						
0.16	146	1.02	-1.3	0.67	-3.2	18.6
0.28	64	0.98	-0.3	0.84	-3.8	11.9
0.37	70	1.00	-0.2	0.84	-1.1	10.5
0.53	72	1.03	-1.4	0.91	-2.3	8.3
0.89	58	0.96	0.5	0.86	-1.2	9.8

References

(1) Karlsson, L.; Zieger, P. Aerosol Particle Number Size Distribution Data Collected during the Arctic Ocean 2018 Expedition; Bolin Centre Database, 2020. https://doi.org/10.17043/oden-ao-2018-aerosol-dmps-1.

(2) Karlsson, L.; Zieger, P. Coarse-Mode Particle Number Size Distribution Data Collected during the Arctic Ocean 2018 Expedition; Bolin Centre Database, 2022. https://doi.org/10.17043/oden-ao-2018-aerosol-coarse-1.

(3) Von der Weiden, S. L.; Drewnick, F.; Borrmann, S. Particle Loss Calculator–a New Software Tool for the Assessment of the Performance of Aerosol Inlet Systems. *Atmos Meas Tech* **2009**, *2* (2), 479–494.

(4) Siegel, K.; Karlsson, L.; Zieger, P.; Baccarini, A.; Schmale, J.; Lawler, M.; Salter, M.; Leck, C.; Ekman, A. M.; Riipinen, I.; Mohr, C. Insights into the Molecular Composition of Semi-Volatile Aerosols in the Summertime Central Arctic Ocean Using FIGAERO-CIMS. *Environ. Sci. Atmospheres* **2021**, *1* (4), 161–175.

(5) Rose, D.; Gunthe, S. S.; Mikhailov, E.; Frank, G. P.; Dusek, U.; Andreae, M. O.; Pöschl, U. Calibration and Measurement Uncertainties of a Continuous-Flow Cloud Condensation Nuclei Counter (DMT-CCNC): CCN Activation of Ammonium Sulfate and Sodium Chloride Aerosol Particles in Theory and Experiment. *Atmospheric Chem. Phys.* **2008**, *8* (5), 1153–1179.

(6) Topping, D.; Barley, M.; Bane, M. K.; Higham, N.; Aumont, B.; Dingle, N.; McFiggans, G. UManSysProp v1. 0: An Online and Open-Source Facility for Molecular Property Prediction and Atmospheric Aerosol Calculations. *Geosci. Model Dev.* **2016**, *9* (2), 899–914.

(7) Zieger, P.; Väisänen, O.; Corbin, J. C.; Partridge, D. G.; Bastelberger, S.; Mousavi-Fard, M.; Rosati, B.; Gysel, M.; Krieger, U. K.; Leck, C. Revising the Hygroscopicity of Inorganic Sea Salt Particles. *Nat. Commun.* **2017**, *8* (1), 1–10.

(8) Markelj, J.; Madronich, S.; Pompe, M. Modeling of Hygroscopicity Parameter Kappa of Organic Aerosols Using Quantitative Structure-Property Relationships. *J. Atmospheric Chem.* **2017**, *74* (3), 357–376.

(9) Lopez-Hilfiker, F. D.; Mohr, C.; Ehn, M.; Rubach, F.; Kleist, E.; Wildt, J.; Mentel, T. F.; Lutz, A.; Hallquist, M.; Worsnop, D. A Novel Method for Online Analysis of Gas and Particle Composition: Description and Evaluation of a Filter Inlet for Gases and AEROsols (FIGAERO). *Atmospheric Meas. Tech.* **2014**, *7* (4), 983–1001.

(10) Huang, W.; Saathoff, H.; Shen, X.; Ramisetty, R.; Leisner, T.; Mohr, C. Seasonal Characteristics of Organic Aerosol Chemical Composition and Volatility in Stuttgart, Germany. *Atmospheric Chem. Phys.* **2019**, *19* (18), 11687–11700.

(11) Mohr, C.; Lopez-Hilfiker, F. D.; Yli-Juuti, T.; Heitto, A.; Lutz, A.; Hallquist, M.; D'Ambro, E. L.; Rissanen, M. P.; Hao, L.; Schobesberger, S.; Kulmala, M.; Mauldin III, R. L.; Makkonen, U.; Sipilä, M.; Petäjä, T.; Thornton, J. A. Ambient Observations of Dimers from Terpene Oxidation in the Gas Phase: Implications for New Particle Formation and Growth. *Geophysical Research Letters*, 2017, *44*, 2958–2966.

(12) Tikkanen, O.-P.; Buchholz, A.; Ylisirniö, A.; Schobesberger, S.; Virtanen, A.; Yli-Juuti, T. Comparing Secondary Organic Aerosol (SOA) Volatility Distributions Derived from Isothermal SOA Particle Evaporation Data and FIGAERO–CIMS Measurements. *Atmospheric Chem. Phys.* **2020**, *20* (17), 10441–10458.

(13) DeCarlo, P. F.; Kimmel, J. R.; Trimborn, A.; Northway, M. J.; Jayne, J. T.; Aiken, A. C.; Gonin, M.; Fuhrer, K.; Horvath, T.; Docherty, K. S. Field-Deployable, High-Resolution, Time-of-Flight Aerosol Mass Spectrometer. *Anal. Chem.* **2006**, *78* (24), 8281–8289.

(14) Leck, C.; Gao, Q.; Mashayekhy Rad, F.; Nilsson, U. Size-Resolved Atmospheric Particulate Polysaccharides in the High Summer Arctic. *Atmospheric Chem. Phys.* **2013**, *13* (24), 12573–12588.

(15) Shantz, N. C.; Leaitch, W. R.; Phinney, L.; Mozurkewich, M.; Toom-Sauntry, D. The Effect of Organic Compounds on the Growth Rate of Cloud Droplets in Marine and Forest Settings. *Atmospheric Chem. Phys.* **2008**, *8* (19), 5869–5887.

(16) Petters, M. D.; Kreidenweis, S. M. A Single Parameter Representation of Hygroscopic Growth and Cloud Condensation Nucleus Activity. *Atmospheric Chem. Phys.* **2007**, *7* (8), 1961–1971.
(17) Chang, R.-W.; Leck, C.; Graus, M.; Müller, M.; Paatero, J.; Burkhart, J. F.; Stohl, A.; Orr, L. H.; Hayden, K.; Li, S.-M. Aerosol Composition and Sources in the Central Arctic Ocean during ASCOS. *Atmospheric Chem. Phys.* **2011**, *11* (20), 10619–10636.
(18) Schmale, J.; Baccarini, A.; Thurnherr, I.; Henning, S.; Efraim, A.; Regayre, L.; Bolas, C.; Hartmann, M.; Welti, A.; Lehtipalo, K. Overview of the Antarctic Circumnavigation Expedition: Study of Preindustrial-like Aerosols and Their Climate Effects (ACE-SPACE). *Bull. Am. Meteorol. Soc.* **2019**, *100* (11), 2260–2283.
(19) Lawler, M. J.; Saltzman, E. S.; Karlsson, L.; Zieger, P.; Salter, M.; Baccarini, A.; Schmale, J.; Leck, C. New Insights Into the Composition and Origins of Ultrafine Aerosol in the Summertime High Arctic. *Geophysical Research Letters*, 2021, *48*, e2021GL094395.
(20) Kammermann, L.; Gysel, M.; Weingartner, E.; Herich, H.; Cziczo, D. J.; Holst, T.; Svenningsson, B.; Arneth, A.; Baltensperger, U. Subarctic Atmospheric Aerosol Composition: 3. Measured and Modeled Properties of Cloud Condensation Nuclei. *J. Geophys. Res. Atmospheres* **2010**, *115* (D4).
(21) Sharma, S.; Andrews, E.; Barrie, L. A.; Ogren, J. A.; Lavoué, D. Variations and Sources of the Equivalent Black Carbon in the High Arctic Revealed by Long-Term Observations at Alert and Barrow: 1989–2003. *J. Geophys. Res. Atmospheres* **2006**, *111* (D14). https://doi.org/10.1029/2005JD006581.
(22) Schulz, H.; Zanatta, M.; Bozem, H.; Leaitch, W. R.; Herber, A. B.; Burkart, J.; Willis, M. D.; Kunkel, D.; Hoor, P. M.; Abbatt, J. P. High Arctic Aircraft Measurements Characterising Black Carbon Vertical Variability in Spring and Summer. *Atmospheric Chem. Phys.* **2019**, *19* (4), 2361–2384.
(23) Weingartner, E.; Burtscher, H.; Baltensperger, U. Hygroscopic Properties of Carbon and Diesel Soot Particles. *Atmos. Environ.* **1997**, *31* (15), 2311–2327.
(24) Hori, M.; Ohta, S.; Murao, N.; Yamagata, S. Activation Capability of Water Soluble Organic Substances as CCN. *J. Aerosol Sci.* **2003**, *34* (4), 419–448.
